# Editorial: Molecular compass to the future - COMPASS 2021

**DOI:** 10.3389/fpls.2023.1257997

**Published:** 2023-08-10

**Authors:** Agnieszka Ludwików, Agata Cieśla, Andrzej Mirosław Pacak

**Affiliations:** ^1^ Laboratory of Biotechnology, Institute of Molecular Biology and Biotechnology, Faculty of Biology, Adam Mickiewicz University, Poznan, Poland; ^2^ Department of Gene Expression, Institute of Molecular Biology and Biotechnology, Faculty of Biology, Adam Mickiewicz University, Poznan, Poland

**Keywords:** doctoral education and training, multidisciplinary collaborations, pollen-mediated gene dispersal, anticancer agents, abiotic stress signaling

Young scientists are now at the forefront, holding the future of science, research, and development in their hands. They are working hard to find the breakthroughs that will address the major challenges of our time. In this Research Topic, which is an outcome of Molecular Compass to the Future - COMPASS Conference 2021, during plenary sessions and workshops young and experienced researchers together have addressed important challenges such as pollen-mediated gene transfer, events in plant abiotic stress signalling and plant-derived anticancer agents. The Research Topic included two reviews that summarise the anticancer activity of plant compounds, one focusing on the cytotoxicity and anticancer activity of latex-bearing plant extracts (Mazur et al.), the other demonstrating the anticancer potential of Solanaceae glycoalkaloids (Winkiel et al.); and two original research, one showed the fitness and foreign protein expression of the hybrid offspring of transgenic and wild soybeans (Liu et al.), the other demonstrate the role of SnRK2.10 in Arabidopsis stress response (Kulik et al.).

Among the compounds with anticancer activity, a group of Solanaceae glycoalkaloids from various Solanaceae and latex-bearing plants appears to be an effective tool in the fight against cancer (Kowalczyk et al., 2022). In their review, Winkiel et al. summarise recent results from studies on the mechanisms of action and anticancer properties of GAs (glycoalkaloids) and their toxicity to cancer cells. Low concentrations of GAs act on a variety of cancer cell lines through a variety of mechanisms. Available results demonstrating antiproliferative and anti-angiogenic activities suggest a potential antitumour effect of GAs on solid tumours. Mazur et al. summarise the available studies on the cytotoxicity and anticancer activity of extracts and individual compounds from latex-bearing plants. In addition, the authors draw attention to the drawbacks of plant-based pharmaceuticals and suggest ways to successfully stabilise plant bioactive compounds. We believe that further research is needed to explore new, more selective, and less toxic cancer therapies and to improve the efficacy of the treatment used.

In recent years, pollen-mediated gene transfer from genetically modified crops has attracted much attention due to environmental and food safety concerns (Chang et al., 2018). Soybean is considered as the most important bean, grown for oil and protein, that is still under pressure to be further developed as a GMO to become more resistant to herbicides, pests, drought, or salt stress. Liu et al. tested the fitness of hybrid progeny between EPSPS (5-enolpyruvylshikimate-3-phosphate synthase) transgenic and wild soybeans to learn more about the fitness of hybrid offspring and wild type soybeans. They found that the fitness of the F1 progeny was significantly lower than that of the parent. Only some fitness traits, including biomass and 100 seed weight, were more pronounced in the hybrid F2 generation. Overall, the results show that the *EPSPS* gene has no fitness cost in soybean.

Abiotic stresses, including salinity, have a significant impact on plant productivity worldwide. Many different effectors are involved in the transduction of stress signals in plants (Peck and Mittler, 2020). Kulik et al. focus on the sucrose non-fermenting-1-related protein kinase 2.10 (SnRK2.10) and WRKY transcription factors. The authors show that salt induces SnRK2.10 activity, which positively regulates the expression of the TFs WRKY33, WRKY40, WRKY46 and WRKY75. However, activation of the SnRK2.10 kinase in response to H_2_O_2_ has the opposite effect. In our opinion, it would be interesting in the future to investigate the mechanism behind these different effects of SnRK2.10 activation on *WRKY* genes expression.

Overall, the articles in this Research Topic show the multidisciplinary nature of the COMPASS conference 2021. We believe that COMPASS has created the platform for young researchers to share research results, receive valuable feedback, and connect with experts from other institutions and disciplines. Events such as these are a good opportunity for young scientists to meet in a friendly environment and to discuss scientific problems freely with fellow scientists and experts. This will allow them to gain confidence in presenting their results at future events.

## Author contributions

AL: Writing – original draft, Writing – review & editing. AC: Writing – review & editing. AP: Writing – review & editing.
